# Methionine deprivation suppresses triple-negative breast cancer metastasis *in vitro* and *in vivo*

**DOI:** 10.18632/oncotarget.11615

**Published:** 2016-08-25

**Authors:** Hyein Jeon, Jae Hwan Kim, Eunjung Lee, Young Jin Jang, Joe Eun Son, Jung Yeon Kwon, Tae-gyu Lim, Sunghoon Kim, Jung Han Yoon Park, Jong-Eun Kim, Ki Won Lee

**Affiliations:** ^1^ Major in Biomodulation, Department of Agricultural Biotechnology and Research Institute for Agriculture and Life Sciences, Seoul National University, Seoul, Republic of Korea; ^2^ Advanced Institutes of Convergence Technology, Seoul National University, Suwon, Republic of Korea; ^3^ Traditional Alcoholic Beverage Research Team, Korea Food Research Institute, Seongnam, Republic of Korea; ^4^ Metabolic Mechanism Research Group, Korea Food Research Institute, Seongnam, Republic of Korea; ^5^ Program in Molecular Medicine, University of Massachusetts Medical School, Worcester, MA, USA; ^6^ Division of Strategic Food Research, Korea Food Research Institute, Seongnam, Republic of Korea; ^7^ Medicinal Bioconvergence Research Center, College of Pharmacy, Seoul National University, Seoul, Republic of Korea; ^8^ Department of Food Science and Nutrition, Hallym University, Chuncheon, Republic of Korea

**Keywords:** methionine, triple-negative breast cancer, metastasis, cancer therapy

## Abstract

Nutrient deprivation strategies have been proposed as an adjuvant therapy for cancer cells due to their increased metabolic demand. We examined the specific inhibitory effects of amino acid deprivation on the metastatic phenotypes of the human triple-negative breast cancer (TNBC) cell lines MDA-MB-231 and Hs 578T, as well as the orthotopic 4T1 mouse TNBC tumor model. Among the 10 essential amino acids tested, methionine deprivation elicited the strongest inhibitory effects on the migration and invasion of these cancer cells. Methionine deprivation reduced the phosphorylation of focal adhesion kinase, as well as the activity and mRNA expression of matrix metalloproteinases MMP-2 and MMP-9, two major markers of metastasis, while increasing the mRNA expression of tissue inhibitor of metalloproteinase 1 in MDA-MB-231 cells. Furthermore, methionine restriction downregulated the metastasis-related factor urokinase plasminogen activatior and upregulated plasminogen activator inhibitor 1 mRNA expression. Animals on the methionine-deprived diet showed lower lung metastasis rates compared to mice on the control diet. Taken together, these results suggest that methionine restriction could provide a potential nutritional strategy for more effective cancer therapy.

## INTRODUCTION

Breast cancer is the second most common form of cancer arising in women in worldwide [1]. Recently, the mortality rate of breast cancer patients has been declining due to early detection methods and improvements in surgery, radiation therapy, chemotherapy, and hormone therapies [2]. However, some patients show resistance of these therapies. Triple-negative breast cancers (TNBC) account for 15-20% of all breast cancers. TNBC refers to an absence of the expression of three major hormone receptors; the estrogen receptor (ER), progesterone receptor (PR), and hormone epidermal growth factor receptor 2 (HER-2). A number of potent receptor-targeting drugs such as tamoxifen and trastuzumab target these receptors, and are therefore ineffective for TNBC patients [3]. TNBC is generally more aggressive, with higher rates of relapse and lower rate of survival in metastatic status [4]. Consequently, patients with TNBC have lower survival rates and a greater risk of the disease relapsing within 5 years of diagnosis. Metastasizing advanced stage of breast cancer remains very difficult to treat, and the ultimate cause of death in many patients is metastasis to a distant site, rather than due to the primary tumor [5, 6]. Therefore, the control of metastasis is a key therapeutic approach for extending overall survival. TNBC patients also have a higher rate of metastatic recurrence (33.9%) compared to other breast cancer patients (20.4%) [7]. Therefore, preventing metastasis of TNBC is an excellent strategy for curing TNBC patient. Adjuvant therapy to prevent metastasis is typically required for the treatment of TNBC.

Cancer cells require larger quantities of nutrient resources such as glucose and amino acids than non-malignant cells, due to their rapid growth and aggressive characteristics. The increased rate of glycolysis exhibited by many tumors cells is referred to as the Warburg effect [8]. Due to the fact that glucose restriction in clinical settings can result in systemic toxicity, dietary amino acid restriction has emerged as a more viable nutritional therapeutic strategy [9]. Limited success has been reported with nutrient deprivation strategies against various cancer cells that are amino acid-dependent. For example, _L_-asparaginase is a chemotherapeutic drug that has been used for the treatment of lymphoblastic leukemia [10, 11]. Methionine is another important amino acid for cancer cell. It is the first amino acid of protein and it is required for protein synthesis, methylation of DNA and polyamine synthesis [12]. Serine starvation also reduces cancer cell survival due to oxidative stress, particularly in cells lacking p53 [13], while tyrosine and phenylalanine restriction have been observed to inhibit B16-BL6 tumor growth and metastasis [14, 15]. Taken together, these results demonstrate proof-of-concept for amino acid restriction as a viable adjuvant therapeutic strategy.

In the present study, we evaluated the effects of amino acid deprivation on the migration and invasion of TNBC cell lines. Among the 10 essential amino acids tested, methionine deprivation caused the strongest inhibitory effect on migration and invasion in these cells. Conditions of methionine deprivation suppressed metastasis-related biomarkers and increased levels of their inhibitors. We also analyzed the effects of methionine restriction on lung metastasis in an orthotopic mouse metastasis model.

## RESULTS

### Methionine deprivation inhibits cell migration and invasion of MDA-MB-231 and Hs 578T cells

**Figure 1:**
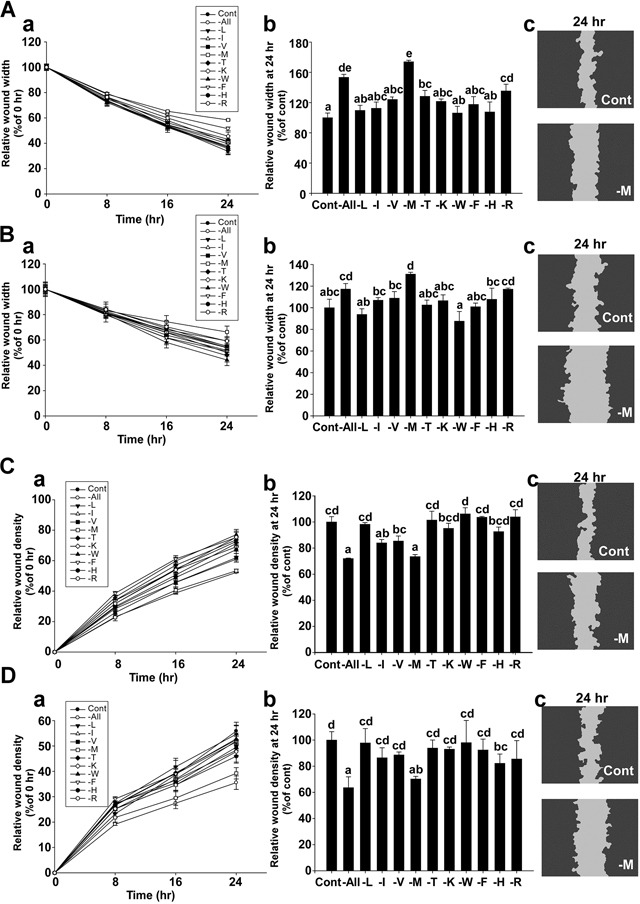
Effect of methionine deprivation on cell migration in MDA-MB-231 and Hs 578T cells **A.** Migration assay results using MDA-MB-231 cells. The relative wound widths of each amino acid-deprived group were compared with the control group over time (a). Comparison of relative wound widths between treatment groups after 24 hours (b). Representative image of a scratch wound mask after 24 hours (c). **B.** Migration assays using Hs578T cells. The relative wound widths of each amino acid-deprived group were compared with the control group over time (a). Comparison of the relative wound widths of each amino acid-deprived and control group after 24 hours (b). Representative image of a scratch wound mask after 24 hours (c). The wound widths were automatically measured using IncuCyte™ software, with the following equation: Wound width $\left(\mu m\right)=\frac{1}{N}\sum w_{i}$ (*N*: vertical line of the scratch wound image, *w*: distance between the edges of the scratch wound mask, *i*: number of 924 horizontal lines of resolution in a scratch wound image). **C.** Invasion assay using MDA-MB-231 cells. The relative wound density of each amino acid-deprived group was compared with the control group over time (a). Relative wound densities for each amino acid-deprived group compared with the control group after 24 hours (b). Representative image of a scratch wound mask after 24 hours (c). **D.** Invasion assay using Hs578T cells. The relative wound density of each amino acid-deprived group was compared with the control group over time (a). Comparison of relative wound densities between treatment groups after 24 hours (b). Representative image of a scratch wound mask after 24 hours (c). The relative wound density (RWD) is a measure of (%) density of the wound region relative to the density of the cell region, which was automatically measured using IncuCyte™ software, with the equation as follows: $\%\text{RWD}\left(t\right)=100\times \frac{\left\{w\left(t\right)-w\left(0\right)\right\}}{\left\{c\left(t\right)-w\left(0\right)\right\}}$ (*w(t)* = Density of wound region at time, t. *c(t)* = Density of cell region at time, t). Data represent the mean values ± S.D. Mean values with letters (a-e) within a graph are significantly different from each other at *p* < 0.05.

To investigate the effect of deprivation of each amino acid in regards to triple negative breast cancer (TNBC) cell migration, MDA-MB-231 and Hs 578T cells were incubated with each amino acid-deprived media formulation for 24 hours. Wound widths at each time point were expressed as the percentage relative to the wound width at 0 hours in each well (Figure 1A(a) and 1B(a)). The relative wound widths of the methionine-deprived group were 174% for MDA-MB-231 (Figure 1A(b) and 131.1% for Hs 578T cells (Figure 1B(b)). The cells of the control group migrated to a greater extent than the cells of the methionine-deprived group (Figure 1A(c) and 1B(c)).

To examine the effect of deprivation of each amino acid on TNBC cell invasiveness, MDA-MB-231 and Hs 578T cells were incubated with each amino acid-deprived media formulation containing BD Matrigel (500 μg/ml) for 24 hours. The relative wound density (RWD) at each time point was expressed as the percentage relative to the wound density at 0 hours in each well (Figure 1C(a) and 1D(a)). The RWD of the methionine-deprived group was 73.5% for MDA-MB-231 (Figure 1C(b)) and 70.2% for Hs 578T cells (Figure 3D(b)). The cells of the control group were more invasive than the cells of the methionine-deprived group (Figure 1C(c) and 1D(c)). Overall, these results indicate that methionine deprivation elicits the strongest inhibitory effect on cell migration and invasion in these cell lines.

### Methionine deprivation has no significant effect on cell viability of MDA-MB-231, Hs 578T, or MCF 10A cells

**Figure 2:**
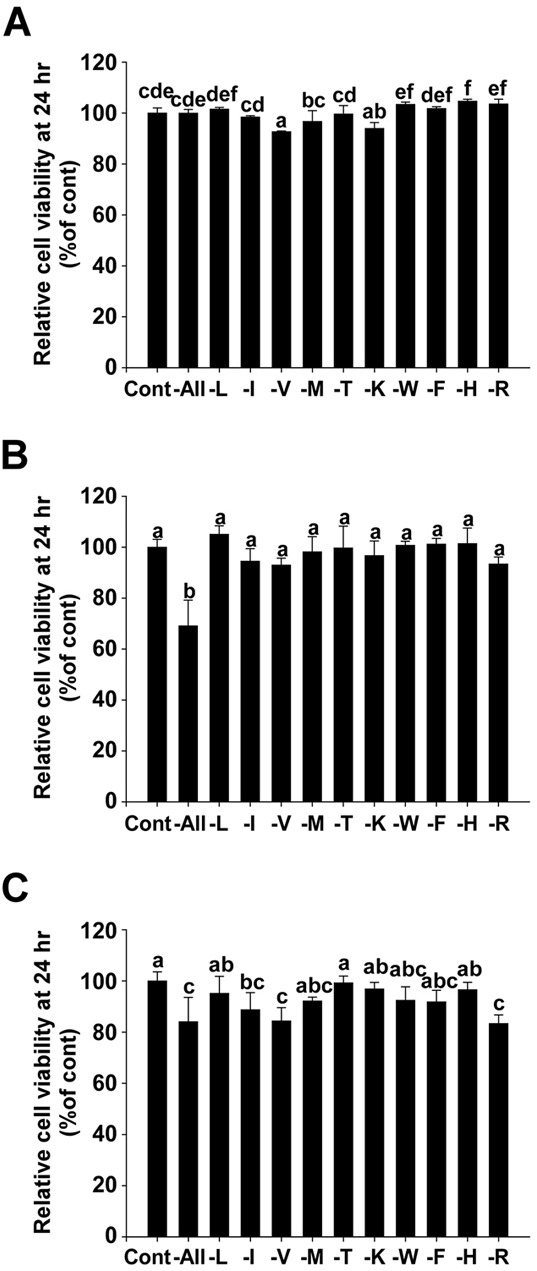
Effect of methionine deprivation on cell viability of MDA-MB-231, Hs 578T cells and the non-invasive breast cell line MCF 10A **A.** Relative cell viability of MDA-MB-231. **B.** Relative cell viability of Hs 578T. **C.** Relative cell viability of MCF 10A. Viability was measured using MTT assay. Data (*n* = 3) represent the mean values ± S.D. Mean values with letters (a-f) within a graph are significantly different from each other at *p* < 0.05.

Methionine deprivation had no significant effect on cell viability for 24 hours in MDA-MB-231 (Figure 2A) and Hs 578T cells (Figure 2B) when assessed using MTT assay. This suggests that the migration and invasion results previously obtained (Figure 1) were a direct result of the inhibition of migration and invasion factors, rather than an indirect effect of proliferative inhibition. In addition, there was no significant difference on cell viability between the methionine-deprived group and the control group for 24 hours when assessed using the normal breast cell line MCF 10A (Figure 2C).

### Methionine deprivation suppresses FAK phosphorylation and activity, in addition to mRNA expression of MMP-2 and MMP-9 in MDA-MB-231 cells

**Figure 3:**
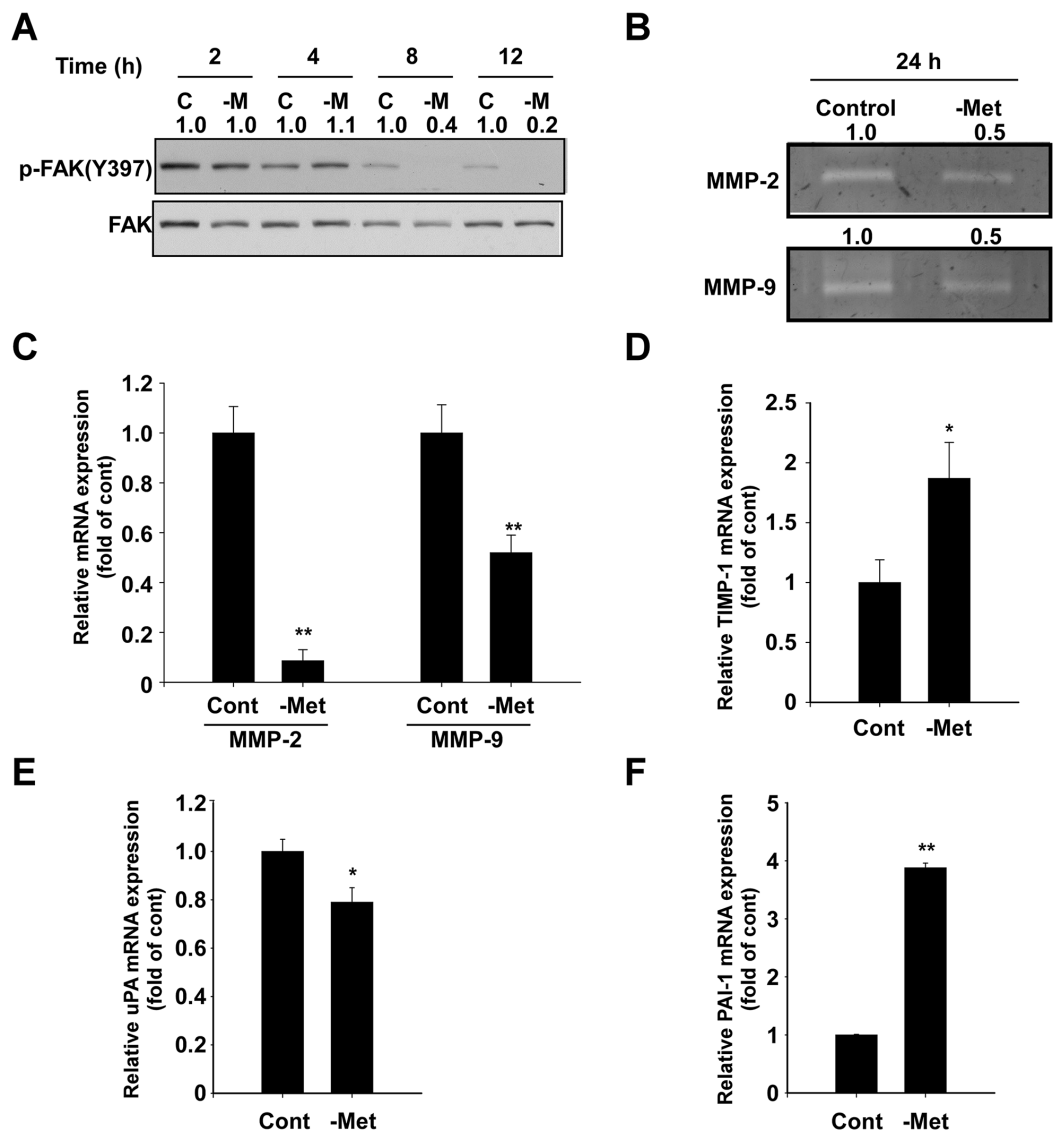
Effect of methionine deprivation on FAK, MMP-2, MMP-9, TIMP-1, uPA, and PAI-1 expression in MDA-MB-231 cells **A.** Methionine deprivation inhibits phosphorylation of FAK at Tyr 397, a major autophosphorylation site, as determined by Western blot. Total FAK was used as a loading control. Cells were incubated in control and methionine-deprived media for the indicated durations and harvested simultaneously. The numbers represent ratio between normal and methionine deprivation media in each time point. Phosphorylation bands are normalized by total band. Band density was measured by the Image J. **B.** MMP-2 and MMP-9 activity was determined by gelatin zymography as described in the Materials and Methods. The numbers represent ratio between normal and methionine deprivation media. The bands are quantified by Image J software. **C.** mRNA levels of MMP-2 and MMP-9 were analyzed by real-time quantitative PCR. Cells were incubated with control and methionine-deprived media for 24 hours and RNA was harvested. **D.** mRNA expression of TIMP-1. **E.** mRNA expression of uPA. **F.** mRNA expression of PAI-1. Data represent the mean values ± S.D. The asterisks (* or **) indicate a significant difference (*p* < 0.05 or *p*< 0.01) between the control group and methionine deprivation group.

We subsequently investigated how methionine deprivation suppresses cell migration and invasion. We first analyzed levels of focal adhesion kinase (FAK), an important protein in cell adhesion, motility and metastasis [16]. Methionine deprivation effectively inhibited phosphorylation of focal adhesion kinase (FAK) after 8 hours (Figure 3A), while also causing a reduction of activity and mRNA expression of MMP-2 and MMP-9, two extracellular matrix-degrading enzymes (Figure 3B and 3C). Tissue inhibitor of metalloproteinase-1 (TIMP-1) mRNA levels were observed to increase in conditions of methionine deprivation (Figure 3D). Furthermore, mRNA levels of urokinase plasminogen activator (uPA) decreased (Figure 3E), while plasminogen activator inhibitor-1 (PAI-1) increased (Figure 3F).

### Methionine deprivation inhibits cell migration and invasion in 4T1 cells

**Figure 4:**
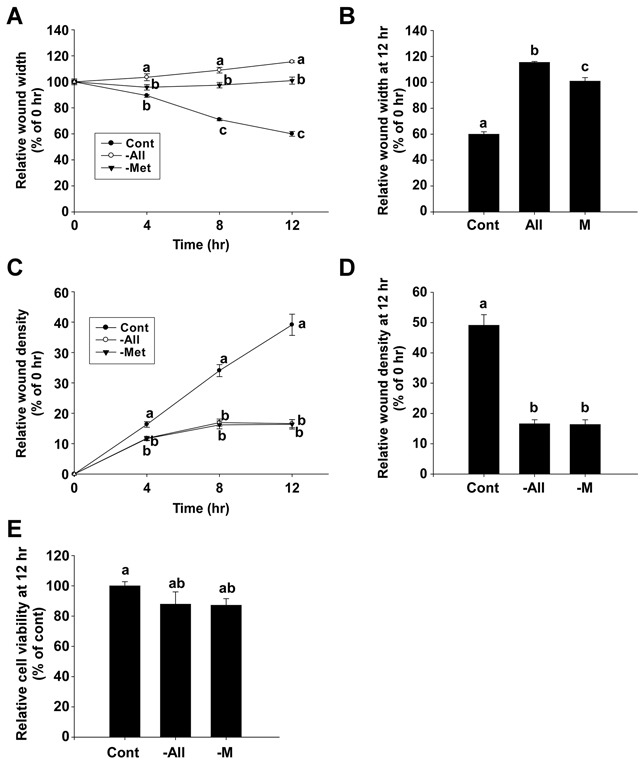
The inhibitory effect of methionine deprivation on migration and invasion of 4T1 cells **A** and **B.** Relative wound widths of 4T1 cells were evaluated by migration assay. **C** and **D.** The relative wound density in 4T1 cells was determined by invasion assay. **E.** Relative cell viability of 4T1 cells. Data represent the mean values ± S.D. Mean values with letters (a-c) within a graph are significantly different from each other at *p* < 0.05.

We next examined the inhibitory effect of methionine deprivation on the migration and invasion of 4T1 mouse triple negative breast cancer cells. The wound width at each time point was expressed as the percentage relative to the wound width at 0 hours in each well (Figure 4A). The relative wound width of the methionine-deprived group at 12 hours was 100.9% compared to a 60% relative wound width for the control group (Figure 4B). The RWD at each time point was expressed as the percentage relative to the wound density of 0 hours in each well (Figure 4C), and the RWD of the methionine-deprived group was 16.4% compared to a 49.1% RWD for the control group (Figure 4D). These inhibitory effects of methionine deprivation were evident within the same time duration that cytotoxicity was absent (Figure 4E).

### Methionine deprivation reduces lung metastasis of 4T1 cells in BALB/c mice

**Figure 5:**
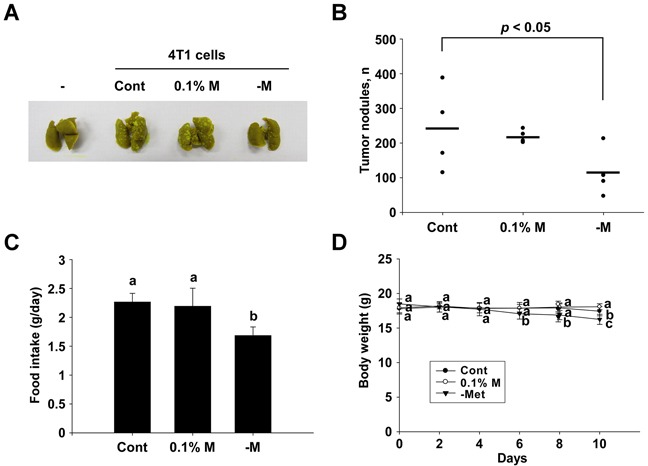
Inhibitory effect of a methionine deprived-diet on lung metastasis in BALB/c mice **A** and **B.** Numbers of tumor nodules detected. **C.** Food intake details for each group. **D.** Mouse body weights. Data (n = 4-5) represent the mean values ± SEM. Mean values with letters (a-c) within a graph are significantly different from each other at *p* < 0.05.

Tail vein injections of 4T1 cells (2 × 10^5^ cells/mouse) to the BALB/c mice caused a development of tumor nodules in the lungs of the mice. An intake of 0.1% methionine in the diet was observed to have no effect, but the intake of a methionine-deprivation diet for 10 days significantly reduced the number of tumor nodules in the lungs (Figure 5A and 5B). The diets were provided ad libitum, and the intake of the methionine deprived-diet (1.69 g/day/mouse) was found to be lower than that of the control diet (2.27 g/day/mouse) (Figure 5C). The body weights of the mice between the control and methionine-deprived group were significantly different after 6 days of injection (Figure 5D).

## DISCUSSION

Breast cancer has the highest incidence among female cancers. Hormonal effects play a pivotal role in breast cancer growth. Endocrine therapy utilizing hormone receptor inhibitor such as tamoxifen is effective in treating breast cancer [17]. However, some types of breast cancer are not responsive to tamoxifen. In tamoxifen-resistant breast cancer, HER-2-targeted therapy is developed for alternative therapy such as trastuzumab [18]. Yet, these therapies do not work in 15∼20% of breast cancer patients [17]. Resistance to drug also develops very easily. The breast cancer which does not respond to endocrine and HER-2-targeted therapy is called as TNBC [4]. TNBC has highly metastatic and aggressive features and there is no effective therapy which results in high mortality. Therefore, new therapeutic strategies are urgently needed for TNBC. In this study, we found methionine restriction inhibits metastasis of TBNC, and it may be a good strategy to prolong lifespan of TNBC patients.

In this study, we employed two human TNBC cell lines, MDA-MB-231 and Hs578T, which are commonly studied human cell lines for treatment of triple negative breast cancer [19]. We also used the 4T1 orthotopic model of TNBC with female BALB/c mice. The tail vein metastasis model with 4T1 cells has established itself as a relevant model for lung metastasis in breast cancer [20]. We screened 10 essential amino acids and observed that methionine restriction conferred the highest inhibitory effect on migration and invasion of human TNBC cells. These inhibitory effects occurred within a short duration after treatment initiated, during which the effect of deprivation was not observed to be cytotoxic, as evidenced by MTT assay for the human TNBC cells and MCF 10A normal breast epithelial cells. Furthermore, the effects of methionine deprivation on migration, invasion and cell viability were confirmed in our 4T1 mouse breast cancer model, and a methionine-deprived diet suppressed metastasis to the lungs of 4T1 cells in BALB/c mice. Although the body weights of methionine-restricted mice were slightly decreased, this is unlikely to be the sole factor responsible for the decrease in metastasis observed. A previous study reported that a methionine-restricted diet negatively impacts the growth of animals, but extends the life span of various rat strains. The study showed that the life span extension effects of low methionine was not due to reduced energy intake, which was also observed to a slight degree [21]. Therefore, the slight weight loss we observed may be a reasonable side effect of a methionine restricted diet.

The Warburg effect refers to the significantly increased need for nutrients and stronger metabolic processes exhibited by cancer cells [8]. In addition to calorie restriction, specific amino acid restriction has been used in the past to treat cancer. Methionine-dependency of malignant cells were first observed in Walker-256 carcinosarcoma-transplanted rodents in 1959 [22]. An absolute methionine-dependency of human, mouse, and rat malignant cells was identified by growth of the cells in methionine-depleted and in homocysteine (the immediate precursor of methionine)-supplemented media (Met^−^ Hcy^+^). In contrast, normal cells were not affected by the Met^−^ Hcy^+^ media [23]. Other studies have suggested that various cancer cells are methionine-dependent, and simple dietary depletion of methionine has been shown to reduce the proliferation of numerous cancer cell lines [9]. For example, methionine starvation has been observed to inhibit tumor growth of PC-3 cells, and a methionine analogue was shown to potentiate these effects [24]. A methionine-restricted diet also suppressed colon cancer development in F344 rats [25]. In addition to the growth inhibitory effects of methionine depletion, it has also been shown to enhance the efficacy of chemotherapeutic agents in refractory cancers and TNBC [26, 27]. Furthermore, methionine restriction is known to extend the life-span of various rat strains, indicating that basic health is not threatened by methionine restriction [21, 28].

Methionine is an essential amino acid necessary for mammal development [9]. Normal cells can convert homocysteine to methionine. However, cancer cells need much more methionine than normal cells do and cannot convert enough methionine they need [29]. Another reason of cancer cells depend on methionine is that methionine is required in polyamine synthesis. Polyamine plays an important roles in cell growth and its level has been associated with colon, lung, prostate skin and breast cancers. Cancer cells cannot produce enough polyamine in methionine deprivation. Especially, methylthioadenosine phosphorylase (MTAP) is involved polyamine synthesis from methionine [12]. Loss of MTAP expression has been observed in many cancer cell lines including TNBC [30]. The cancers which lose MTAP expression required methionine and cannot grow in methionine deprivation [30–32].

Many studies have showed inhibition of growth of cancer cell by methionine deprivation [9]. In our knowledge, we showed for the first time methionine-deprivation reduced TNBC migration *in vitro* and *in vivo* in this study. FAK is required for the signaling pathway initiated by the interaction between ECM proteins and integrins which induce cell migration [33]. We changed normal and methionine free medium. In normal medium, the highest phosphorylation of FAK was shown and the phosphorylation of FAK decreased in a time-dependent manner. In the methionine free medium, the phosphorylation of FAK also decreased in a time-dependent manner but much more than that of normal medium. In the same time points, phosphorylation of FAK was decreased by methionine deprivation. FAK regulates migration related proteins such as MMP-2, MMP-9, TIMP-1, uPA, and PAI-1 [34]. MMP-2 and MMP-9 are gelatinase which degrade ECM and make cell free to move [35]. TIMP-1 inhibits MMPs activity [36]. uPA is a serine protease that can initiate proteolytic cascades, which result in remodeling of basement membrane and ECM allowing cells to move [37]. PAI-1 inhibits uPA [38]. Methionine deprivation inhibited MMP-2, MMP-9 and uPA, and increased TIMP-1 and PAI-1. Therefore, methionine is required for TNBC migration by regulating FAK.

Taken together, these observations suggest that methionine restriction should be regarded as a possible adjuvant therapeutic strategy for TNBC treatment. Underlying mechanism how methionine deprivation inhibits phosphorylation FAK remains to be elucidated. Clinical trial is also needed to validate methionine deprivation is an effective strategy for TNBC treatment.

## MATERIALS AND METHODS

### Reagents

Dulbecco's modified eagle medium (DMEM) and the amino acid-deprived DMEM formulations were purchased from Welgene (Daegu, Korea). Fetal bovine serum (FBS), mitomycin C, recombinant human epithelial growth factor and insulin solution were obtained from Sigma-Aldrich (St.Louis, MO). Dialyzed FBS (dFBS), HEPES, and anti-FAK (pY397) antibodies were purchased from Life Technologies (Grand Island, NY). The anti-FAK antibody was obtained from Millipore (Billerica, MA). Penicillin-Streptomycin (pen/strep) solution was purchased from Mediatech, Inc. (Manassas, VA). Matrigel was obtained from Corning (Corning, NY) and protein assay reagent kits were obtained from Bio-Rad Laboratories (Hercules, CA). A10021B and Modified Diets were obtained from Central Lab. Animal, Inc. (Seoul, Korea; details in Supplementary Table S1).

### Cell culture

The human triple-negative breast cancer cell lines MDA-MB-231 and Hs 578T, and mouse triple negative breast cancer 4T1 cells were maintained in DMEM containing 10% (v/v) FBS and 1% (v/v) pen/strep at 37°C and 5% CO_2_. Human breast epithelial MCF 10A cells were maintained in DMEM with 10% (v/v) FBS, 1% (v/v) pen/strep, 15 mM hepes buffer, 10 μg/ml insulin and 20 ng/ml EGF. Each amino acid-deprived DMEM formulation was supplemented with 10% (v/v) dFBS and 1% (v/v) pen/strep.

### Migration assay

In order to analyze the migratory capacity of the TNBC cells, Essen ImageLock 96-well plates (Essen Instruments, Hertfordshire, UK) were coated with BD Matrigel (100 μg/ml culture media, BD Biosciences, San Jose, CA) overnight. MDA-MB-231 (4 × 10^4 cells/well), Hs 578T (2 × 10^4 cells/well) and 4T1 (3 × 10^4 cells/well) were then seeded on the Essen ImageLock plates. The cells reached confluence after 24 hours, and mitomycin C (25 μg/ml serum free media) was treated 30 minutes prior to media change after the cells were washed with PBS. A single wound was made across each well automatically using a Wound Maker 96 Tool (Essen Instruments). The cell debris was washed with PBS and each amino acid-deprived media was applied as a treatment condition. Wound images were monitored with an IncuCyte Live-Cell Imaging System and software (Essen Instruments) and data were analyzed by custom algorithms in the IncuCyte software package.

### Invasion assay

In order to determine the invasiveness of TNBC cells, Essen ImageLock 96-well plates (Essen Instruments) were coated with BD Matrigel (100 μg/ml culture media, BD Biosciences, San Jose, CA) overnight. MDA-MB-231 (4 × 10^4 cells/well), Hs 578T (2 × 10^4 cells/well) and 4T1 (3 × 10^4 cells/well) were then seeded on the Essen ImageLock plates. After 24 hours, the cells reached confluence and a single wound was made across each well automatically using a Wound Maker 96 Tool (Essen Instruments). The cell debris was washed with PBS and each amino acid-deprived media containing BD Matrigel (500 μg/ml) was applied as a treatment condition. Wound images were monitored with the same IncuCyte Live-Cell Imaging System and software (Essen Instruments) and data were analyzed in terms of relative wound density (RWD) calculated by custom algorithms in the IncuCyte software package.

### Cell viability assay

In order to assess cell viability, MDA-MB-231 (4 × 10^4 cells/well), Hs 578T (2 × 10^4 cells/well) and 4T1 (3 × 10^4 cells/well) and MCF 10A (2 × 10^4 cells/well) cells were seeded into 96-well plates. After 24 hours, the wells were treated with each amino acid-deprived media formulation and incubated for 24 hours. MTT [3-(4,5-dimethylthiazol-2-yl)-2.5-diphenyltetrazolium bromide] solution (final concentration: 0.5 mg/ml) was added to each well, and the cells were incubated for 1 hour. The dark formazan crystals that were formed by the intact cells were dissolved in dimethyl sulfoxide, and the absorbance at 570 nm was measured with a microplate reader. The results are expressed as percent MTT reduction relative to the absorbance of the control cells.

### Western blot analysis

After MDA-MB-231 cells were cultured in 6-cm dishes for 24 hours, the cells were incubated in either control media or methionine-deprived media for 2, 4, 8, or 12 hours. The cells were washed with cold PBS and harvested, before the protein concentration was measured using a protein assay reagent kit as described by the manufacturer. The proteins were separated electrophoretically using a 10% SDS-polyacrylamide gel and transferred onto an Immobilon P membrane (Merck Millipore, Billerica, MA). The membranes were blocked in 5% fat-free milk for 1 h, and then incubated with a specific primary antibody at 4°C overnight. Protein bands were visualized using a chemiluminescence detection kit (GE Healthcare, London, UK) after hybridization with a HRP-conjugated secondary antibody (Life Technologies). The bands were quantified using the Image J software program ((National Institutes of Health, Bethesda, MD)).

### Gelatin zymography

After MDA-MB-231 and Hs 578T cells were cultured in 6-cm dishes for 24 hours, the cells were incubated in either control media or methionine-deprived media for 24 hours. The cells were harvested on ice, and then centrifuged at 18,620 g for 10 min. Protein concentration was measured using a protein assay reagent kit as described by the manufacturer. The proteins were separated electrophoretically using a 12% polyacrylamide gel in the presence of gelatin (0.1% w/v) as a substrate for matrix metalloproteinases (MMPs). The protein samples were mixed with loading buffer [10% SDS, 25% glycerol, 0.25 M Tris (pH 6.8) and 0.1% bromophenol blue], and then run on a 12% SDS-PAGE gel without denaturation. The gel was then washed with renaturating buffer (Life Technologies) for 1 h at room temperature and incubated for 24 h at 37°C in developing buffer (Life Technologies). After the enzyme reaction, the gel was stained with 0.5% Coomassie brilliant blue in 10% acetic acid. The bands were quantified using the Image J software program.

### Real-time quantitative PCR

MDA-MB-231 cells were incubated in either control media or methionine-deprived media for 24 hours and harvested using RNAiso Plus (Takara Bio Inc., Shiga, Japan). RNA was quantified using a NanoDrop ND-2000 spectrophotometer (Thermo Fisher Scientific, Waltham, MA). After reverse transcription with oligo-dT primers using a PrimeScript™ 1^st^ strand complementary DNA (cDNA) synthesis kit (Takara Bio Inc.), real-time quantitative RT-PCR was conducted using IQ SYBR (Bio-Rad Laboratories) and 2 μl of cDNA in triplicate with 18s rRNA as an internal control. Prior to PCR amplification, the primers were denatured at 95°C for 3 min. The amplification program consisted of 44 cycles at 95°C for 10 sec, 60°C for 30 sec, and 72°C for 30. PCR was performed using a CFX Connect™ Real-Time PCR Detection System (Bio-Rad Laboratories, Hercules, CA). cDNA was amplified using the following primers: MMP-2 forward (5′-TGG CAA GTA CGG CTT CTG TC-3′); MMP-2 reverse (5′-TTC TTG TCG CGG TCG TAG TC-3′); MMP-9 forward (5′-GCA CGA CGT CTT CCA GTA CC-3′); MMP-9 reverse (5′-CAG GAT GTC ATA GGT CAC GTA GC-3′); TIMP-1 forward (5′-TGC GGA TAC TTC CAC AGG TC-3′); TIMP-1 reverse (5′-GCA TTC CTC ACA GCC AAC AG-3′); TIMP-2 forward (5′-AAG AGC CTG AAC CAC AGG TA-3′); TIMP-2 reverse (5′-GAG CCG TCA CTT CTC TTG AT-3′); u-PA forward (5′-AGC CCT GCC CTG AAG TCG TTA-3′); u-PA reverse (5′-CAG GGC ATC TCC TGT GCA TG-3′); PAI-1 forward (5′-CTC CTG GTT CTG CCC AAG TT-3′); pai-1 reverse (5′-GAG AGG CTC TTG GTC TGA AAG-3′) ; 18s rRNA forward (5′-GTA ACC CGT TGA ACC CCA TT-3′); 18s rRNA reverse (5′-CCA TCC AAT CGG TAG TAG CG-3′). Relative quantitation was performed using the comparative ΔΔCt method following the manufacturer's instructions.

### Animals

Eight-week-old female BALB/c mice were purchased from Orient Bio Inc. (Seongnam, Korea). Animals were acclimated for 5 days prior to the study and had free access to food and water. All experimental protocols were approved by the Institutional Animal Care and Use Committee (Case Number: SNU-141006-4) of Seoul National University, Seoul, Korea. The animals were housed in climate-controlled quarters (24°C at 50% humidity) with a 12 h light/12 h dark cycle.

### *In vivo* lung metastasis experiments

4T1 cells were harvested and diluted in sterile PBS. The cells (2 × 10^5 cells/mouse) were injected into the tail veins of nine-week-old female BALB/c mice. On the day of the injection, the mice were divided randomly into 3 treatment groups and diets were modified accordingly with the following: 1) control diet, 2) 0.1% methionine containing diet, 3) methionine-deprived diet. The food and water were supplied ad libitum. Ten days after the injection, all mice were sacrificed and the lungs and livers were isolated and weighed. The lungs were fixed in Bouin's solution (Sigma-Aldrich, St.Louis, MO) to quantify tumor nodules.

### Statistical analysis

Statistical analysis was performed using one-way ANOVA followed by Duncan's Multiple Range Test, and *p* values of less than 0.05 were considered statistically significant.

## SUPPLEMENTARY MATERIAL TABLE





## Abbreviations

TNBC: triple-negative breast cancers
MMPs: matrix metalloproteinases
FAK: focal adhesion kinase
TIMP-1: tissue inhibitor of metalloproteinase-1
uPA: urokinase plasminogen activator
PAI-1: plasminogen activator inhibitor-1
RWD: relative wound density
