# Supplementary material for: Methionine deprivation suppresses triple-negative breast cancer metastasis in vitro and in vivo
Source: Oncotarget. 2016 Aug 25;7(41):67223–34. doi: 10.18632/oncotarget.11615 (PMC5341870; doi:10.18632/oncotarget.11615)
Supplement: Supplementary file 1 [file oncotarget-07-67223-s001.pdf]

Methionine deprivation suppresses triple-negative breast cancer metastasis *in vitro* and *in vivo*

SUPPLEMENTARY TABLE

Supplementary Table S1: A10021B and Modified Diets. A10021B and Modified Diets were obtained from Central Lab Animal. A10021B is based on Hirakawa et al [14].

See Supplementary File 1
