# Supplementary material for: Methionine deprivation suppresses triple-negative breast cancer metastasis in vitro and in vivo
Source: Oncotarget. 2016 Aug 25;7(41):67223–34. doi: 10.18632/oncotarget.11615 (PMC5341870; doi:10.18632/oncotarget.11615)

**Supplement Table 1**

**Table 1. A10021B and Modified Diets.** A10021B and Modified Diets were obtained from Central Lab Animal. A10021B is based on Hirakawa *et al* [14].

**
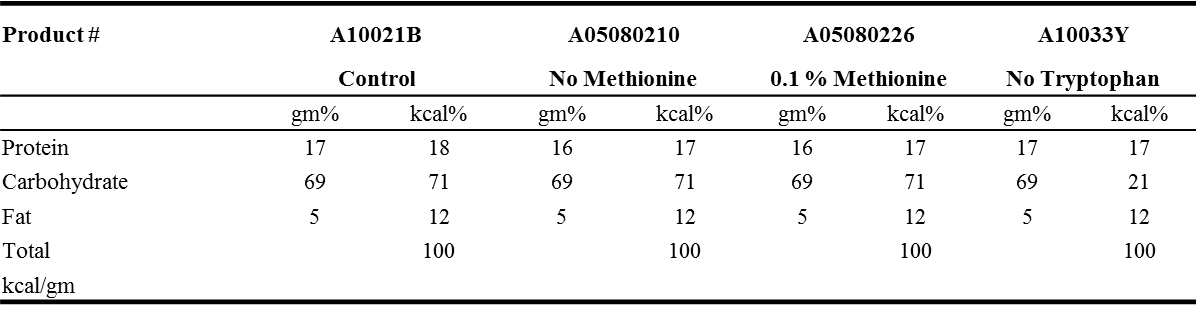
**

**Table 1. continue**

**
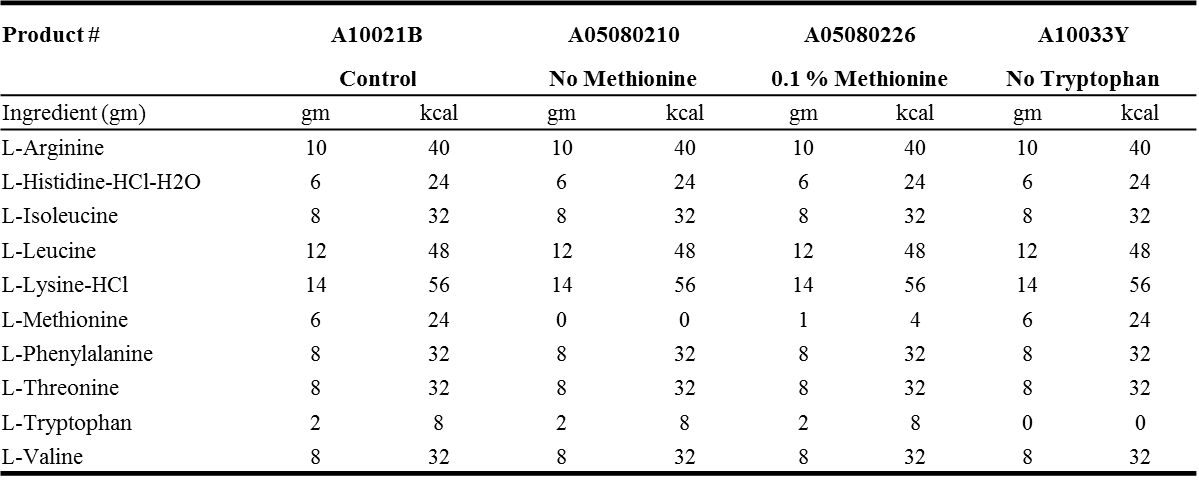
Table 1. continue**

**
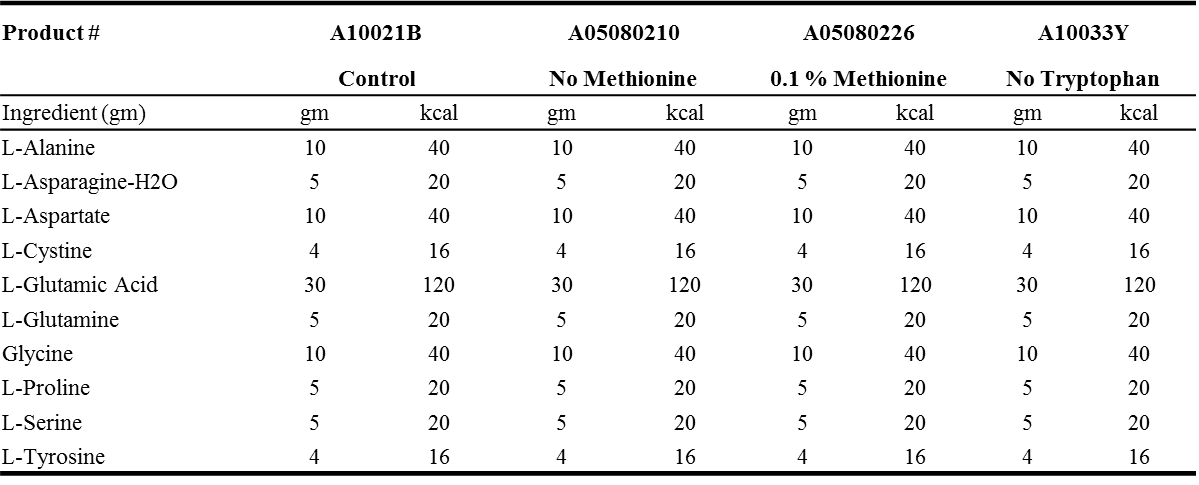
**

**Table 1. continue**


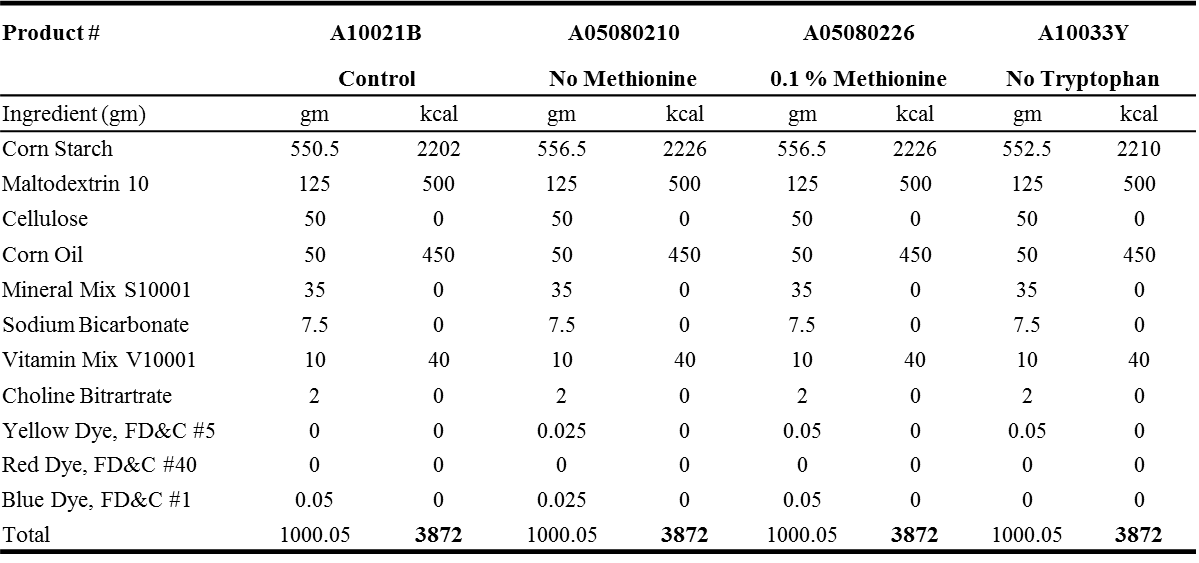

Supplement: Supplementary file 2 [file oncotarget-07-67223-s002.docx]
